# Potential for Using Acetic Acid Plus Pear Ester Combination Lures to Monitor Codling Moth in an SIT Program

**DOI:** 10.3390/insects7040068

**Published:** 2016-11-25

**Authors:** Gary J. R. Judd

**Affiliations:** Summerland Research and Development Centre, Agriculture and Agri-Food Canada, Box 5000, 4200 Hwy 97, Summerland, BC V0H 1Z0, Canada; Gary.Judd@AGR.GC.CA; Tel.: +1-250-494-7711

**Keywords:** *Cydia pomonella*, sterile insect technique, mating disruption, bisexual lures, female monitoring, sterile overflooding ratios

## Abstract

Studies were conducted in commercial apple orchards in British Columbia, Canada, to determine whether lures combining ethyl-(*E*,*Z*)-2,4-decadienoate, pear ester (PE), with either acetic acid (AA) or sex pheromone, (*E*,*E*)-8,10-dodecadien-1-ol (codlemone), might improve monitoring of codling moth, *Cydia pomonella* (L.), in an area-wide programme integrating sterile insect technology (SIT) and mating disruption (MD). Catches of sterile and wild codling moths were compared in apple orchards receiving weekly delivery of sterile moths (1:1 sex ratio) using white delta traps baited with either AA or PE alone, and in combination. Sterile and wild codling moths responded similarly to these kairomone lures. For each moth sex and type (sterile and wild), AA-PE lures were significantly more attractive than AA or PE alone. Bisexual catches with AA-PE lures were compared with those of commercial bisexual lures containing 3 mg of codlemone *plus* 3 mg of PE (Pherocon CM-DA Combo lure, Trécé Inc., Adair, OK, USA), and to catches of males with standard codlemone-loaded septa used in SIT (1 mg) and MD (10 mg) programmes, respectively. CM-DA lures caught the greatest number of sterile and wild male moths in orchards managed with SIT alone, or combined with MD, whereas AA-PE lures caught 2–3× more females than CM-DA lures under both management systems. Sterile to wild (S:W) ratios for male versus female moths in catches with AA-PE lures were equivalent, whereas in the same orchards, male S:W ratios were significantly greater than female S:W ratios when measured with CM-DA lures. Male S:W ratios measured with CM-DA lures were similar to those with codlemone lures. CM-DA and codlemone lures appear to overestimate S:W ratios as measured by AA-PE lures, probably by attracting relatively more sterile males from long range. Using AA-PE lures to monitor codling moths in an SIT programme removes fewer functional sterile males and reduces the need for trap maintenance compared with using codlemone lures. AA-PE lures allow detection of wild female moths that may measure damage potential more accurately than detection of wild males. The short-range activity of AA-PE lures compared with that of codlemone-based lures appears to improve the ability to measure S:W ratios and the impact of SIT on population control near the site where wild moths are trapped.

## 1. Introduction

During the 1990s, several area-wide programmes to manage codling moth, *Cydia pomonella* (L.) (Lepidoptera: Tortricidae), were implemented in commercial apple production areas of western North America [1]. Whereas pheromone-based mating disruption (MD) was the basis of these programmes in the United States [2], in Canada, the area-wide pest management paradigm was founded on previously developed sterile insect technology (SIT). The Okanagan–Kootenay Sterile Insect Release (SIR) Program was initiated in 1992 [3], in an effort to eradicate populations of codling moth from montane fruit-growing valleys of southern British Columbia (BC), Canada. When eradication proved unfeasible, the Canadian SIR Program revised its objective, and area-wide suppression to keep damage below economically acceptable levels became its new target [4,5]. This Canadian SIR Program currently uses an integrated approach to achieve its objective by using insecticides and/or MD in high-risk orchards as supplements to the area-wide SIT programme [6,7].

Effective measurement of relative densities and seasonal flight patterns with pheromone traps has been an important component of codling moth management for over 40 years [3,8,9]. SIT programmes have an additional need to estimate the ratio of sterile (S) and wild (W) insects in mating populations. Under an SIT programme, wild insect populations are only suppressed when sterile insects outnumber fertile insects in the mating population by some critical ratio, often called the S:W overflooding ratio [10]. For codling moth SIT, the target S:W ratio is 40 sterile insects for every wild insect [11]. Operationally, these S:W ratios are measured using male catches in pheromone traps [3,12].

Using male trap catches as a proxy to measure the control effort of a codling moth SIT programme comes with many assumptions. (1) Sterile and wild populations are assumed to be adequately mixed with a similar spatial population structure; (2) sterile and wild moths have an equal probability of being caught; (3) sterile and wild moths have similar behavioural responses to trap lures; and (4) S:W ratios based on male catches reflect actual S:W mating ratios among wild females. These assumptions have never been explicitly tested. We do know, however, that catches of sterile moths is poor in spring, when air and ground temperatures are cool [12], and pheromone traps grossly overestimate actual S:W mating ratios [13,14], perhaps because sterile moths are more responsive to synthetic pheromone lures than are wild moths [13].

Ideally, an SIT programme might measure wild female density, assess the females’ mating status, and directly determine if mated females are infertile, that is, mated to sterile males in the proper ratio. This seems ideal because the density of fertile female insects—the source of eggs and feeding larvae—should correlate more directly with damage potential than the density of male insects. Trapping female codling moths with synthetic lures is clearly a first step towards an ideal sampling tool, and much has been done in this regard since the SIR Program began in 1992 [3]. Ethyl-(*E*,*Z*)-2,4-decadienoate—pear ester (PE), as it is commonly known—is a host-plant kairomone that attracts both male and female codling moth [15]. Action thresholds based on female moth catch or total moth catch using PE lures have been developed [16]. Combining PE with codling moth sex pheromone (codlemone) increases catches of male codling moths [17], and when acetic acid (AA) is combined with PE, catches of female codling moth are increased over catches with PE alone [18]. Both of these PE-based combination lures can improve monitoring of codling moth in orchards treated with sex pheromones for MD [19,20,21].

The availability of a commercial codlemone *plus* PE combination lure (Pherocon CM-DA Combo, Trécé Inc., Adair, OK, USA) and more recent availability of commercial AA lures (Trécé Inc.) has induced many apple growers in western USA to use PE-based lures in their MD programmes [22]. Whether bisexual combination lures based on PE are useful for monitoring codling moths in an SIT programme or measuring its performance by estimating S:W ratios has yet to be assessed. The first step in this assessment is determining whether sterile and wild codling moths exhibit similar responses to these lures in the field. While mass-reared codling moths from the Canadian SIR Program responded to AA, PE, and their combination in flight-tunnel assays [18], these insects had not received sterilizing radiation, nor were they handled using standard SIR Program protocols known to affect pheromone trap catches [14]. The current study has three objectives. (1) To compare the relative catches of sterile and wild codling moths with various bisexual lures in apple orchards managed by the SIR Program; (2) to compare catches of sterile and wild moths with these lures in orchards receiving sterile moths *plus* commercial MD treatments; and (3) to compare and contrast the ratios of sterile and wild moths based on seasonal catches of males and females alone, as well as combined, when using different lures under SIT and SIT + MD management programmes.

## 2. Materials and Methods

### 2.1. Sterile Moth Treatments

Detailed methods used by the Okanagan–Kootenay SIR Program to produce, mark, irradiate, and handle sterile codling moths were described previously [14]. Each test orchard in this study received a single weekly delivery of these mixed-sex (1:1 ratio) sterile codling moths at a rate of 4000 ha^−1^. Moths were evenly distributed throughout all test orchards using an all-terrain vehicle (ATV) equipped with a front-mounted hopper and fan unit that gently dispensed moths onto the ground beneath trees [23]. Starting at one edge of each orchard, the ATV was driven up and down rows making one pass at each 30 m interval. Delivery of sterile moths began 1 May and ceased 31 August each year.

### 2.2. Codling Moth Monitoring Techniques Used by Canadian SIR Program

The Canadian SIR Program monitors adult codling moth in pome fruit orchards using Pherocon 1-CP, sticky wing traps baited with red rubber septa containing 1 mg of codlemone [3]. Orchards that receive supplementary MD treatments are monitored using red rubber septa loaded with 10 mg of codlemone [6,24]. The SIR Program manufactures its own traps and lures. Codling moth traps are deployed at 1 trap ha^−1^, and every hectare of pome fruit orchard in the Okanagan and Similkameen Valleys of southern BC has been monitored since 1992. Traps are deployed by 1 May and checked weekly until 31 August each year, with pheromone lures changed on a three-week rotation and sticky bottoms replaced as required.

### 2.3. Experiment 1

One season-long experiment was conducted to assess the relative catches of sterile and wild codling moths with kairomone components of bisexual lures. This study was conducted in a 4 ha square-shaped apple orchard containing a 4.6 m high mixture of Spartan and McIntosh cultivars planted using a 6.1 × 6.1 m tree-row spacing, in west Kelowna, BC (49.8204° N, −119.6311° W, elevation: 400 m). In this experiment, catches of sterile and wild codling moths in large white delta traps (Trécé Inc., Adair, OK, USA) baited with one of four lure treatments were compared: a blank control, acetic acid (AA), pear ester (PE), and an AA-PE combination. Acetic acid was dispensed from 15 mL polypropylene bottles (Nalg-Nunc International, Rochester, NY, USA). Each bottle contained 10 mL of glacial acetic acid applied to two cotton balls placed in the bottom of each bottle. Volatilised AA was released through a 3 mm diameter hole drilled in each bottle lid. Pear ester (ethyl-(*E*,*Z*)-2,4-decadienoate) was dispensed from grey halobutyl rubber septa (West Co., Lyonville, PA, USA). Each septum was pre-extracted with methylene chloride and loaded with a 200 µL solution of methylene chloride containing 1 mg of pear ester and 10 mg of the antioxidant butylated hydroxytoluene (BHT). All chemicals and solvents were supplied in 99% purity by the Sigma-Aldrich Chemical Co. (St. Louis, MO, USA).

The AA-PE combination lure consisted of an upright AA bottle dispenser and a PE-loaded septum attached to either end of a 10 cm long wire bent into the shape of a horseshoe, such that the tops of two devices were separated by 1 cm in the same horizontal plane [18]. Wires holding these combination lures were pinned to the inside roof of delta traps and positioned in the geometric centre of each trap.

Experimental traps were deployed using a 4 × 4 Latin square design with four replicates of each of the four lure treatments. Each trap was attached by wire to one end of a 2 m long bamboo pole and hooked over a branch near the top of the tree canopy. Traps were deployed using 10 m spacing between traps within a linear array (block), and blocks of traps were separated by 20 m in a grid pattern. Traps were hung in the middle of the orchard ca. 35 m from any SIR Program pheromone traps, and were deployed on 1 June and checked weekly until 31 August, 2008. All sticky-trap liners were collected weekly and returned to the laboratory, where moths were separated by sex and then identified as either wild or sterile, based on the presence of an internal red dye in the latter [14]. All trap locations remained constant throughout the season, and all lures were replaced every three weeks.

This test orchard was also monitored by SIR Program staff using four 1 mg codlemone-baited wing traps (SIR Program, Osoyoos, BC, USA). It was sprayed with insecticides as needed, but received no MD treatment. SIR traps were deployed as standard near the edges and corners of the test block. Seasonal data from SIR Program pheromone traps were not included in our treatment comparisons but were provided by SIR staff for calculating and comparing seasonal S:W ratios.

### 2.4. Experiment 2

Experiments 2 and 3 were conducted to measure the relative responses of sterile and wild codling moths to various lures in apple orchards receiving a pheromone MD treatment compared to their responses in apple orchards receiving no pheromone treatment. In experiment 2, the SIR Program applied Isomate^®^-CM/LR TT (Shin-etsu Chemical Company, Tokyo, Japan), at a density of 750 dispensers ha^−1^, to a 12.5 ha contiguous test area of mixed apple varieties in east Kelowna (49.8738° N, −119.4436° W, elevation: 432 m) that was immediately adjacent to a 17 ha area of apples that received no MD. Both areas received the standard weekly delivery of sterile moths, as described above. Catches in white delta traps baited with either an AA-PE lure or a CM-DA Combo lure (Trécé Inc., Adair, OK, USA) were compared with catches using codlemone lures used in SIT-only (1 mg) and SIT + MD-treated orchards (10 mg), respectively. The CM-DA lure is a grey rubber septum loaded with 3 mg of codlemone *plus* 3 mg of PE [21]. For experiments 2 and 3, the AA-PE lure was modified by combining AA and PE into a single 8 mL propylene bottle and eliminating the septum. Each bottle with a 3 mm diameter hole in its lid contained two small cotton balls and a 5 mL load of an acetic acid plus PE mixture (240:1 µL *v:v*) with no BHT. This lure contained 18.8 mg of PE.

In both the SIT and SIT + MD-treated areas, three lure treatments (AA-PE, CM-DA, and codlemone) were deployed using a randomised block design, where an SIR Program standard codlemone-baited trap location was centred within a linear set (block) of the three different lure treatments. Traps within sets were spaced 5 m apart and each set of traps within each area was spaced at least 100 m apart. The narrow trap-spacing within blocks was used to deliberately place lures in direct competition with each other and to ensure they were sampling the same localised wild populations. One set of experimental lures was hung at each of five SIR Program standard trap locations within the SIT-only and SIT + MD-treated areas, respectively. At these five replicate locations, the SIR Program wing traps and pheromone lures were replaced with delta traps baited with our own pheromone lures. Grey rubber septa were pre-extracted as above, and loaded with either 1 or 10 mg of codlemone (99% isomerically pure *E*,*E*-8,10-dodecadien-1-ol, Shin-etsu, Fine Chemicals Division, Tokyo, Japan) dissolved in 200 µL of methylene chloride, for use in orchards managed by SIT or SIT + MD, respectively. All experimental traps were deployed on 1 June and checked and serviced weekly until 31 August 2009. All trap locations remained constant throughout the season, and all lures were replaced every three weeks.

### 2.5. Experiment 3

Catches of sterile and wild codling moths with AA-PE, CM-DA, and a broader range of codlemone-loaded lures were compared in several individual apple orchards being managed by SIT or SIT + MD using a new MD formulation and treatment level in south Kelowna (49.8389° N, −119.4436° W, elevation: 426 m). In experiment 3, the SIR Program applied Isomate^®^-CM-Flex (Shin-etsu Chemical Company) at a density of 1000 dispensers ha^−1^ to four apple orchards as part of an integrated SIT + MD strategy for high-risk orchards. These four orchards ranged in size from 3.8 to 4.5 ha and all were high-density plantings (1 × 3 m tree-row spacing) of mixed apple varieties on dwarfing rootstocks. In each SIT + MD orchard, we compared catches in delta traps baited with AA-PE and CM-DA lures against catches with 0.1, 1.0, and 10 mg codlemone-loaded grey septa lures. Each SIT + MD apple orchard was paired with an adjacent SIT apple orchard located on the same legal property. The SIT orchards ranged in size from 3.1 to 6.1 ha and all were high-density plantings of similar varieties to the SIT + MD orchards. In these SIT-only orchards, we compared catches in delta traps baited with AA-PE and CM-DA lures against 0.1 and 1.0 mg codlemone-loaded grey septa lures.

In experiment 3, lure treatments tested within each orchard were deployed using the same randomised block design as experiment 2. One set (block) of experimental traps was hung at each of three SIR Program standard trap locations within each of the four SIT-only and SIT + MD-treated orchards. There were 12 traps in total for each lure treatment within the SIT + MD-treated and SIT-only orchard categories, respectively. Again, traps within sets were spaced 5 m apart to place lures in competition for localised wild populations, but each set of 4–5 traps within an orchard was spaced at least 50 m apart to sample different parts of the orchard. All experimental traps were deployed 1 June and checked and serviced as before until 31 August 2010.

### 2.6. Data Analyses

Trapping data from all experiments were first analysed by an analysis of variance (ANOVA), and means were compared using Tukey’s honest significant difference (HSD) multiple-comparison test following significant ANOVA [25]. Experiment 1 employed a Latin square design, and data were analysed using a three-way ANOVA with one fixed effects factor (lure treatments) and two random factors (rows and columns of the square) as described by [25]. Separate analyses were performed on mean (*n* = 4) season-long cumulative catches of males, females, and total catches for both sterile and wild moths. In experiment 2, the lure treatments within the SIT and SIT + MD areas were compared separately using two-way randomised block ANOVAs, where sets of traps are replicate blocks (*n* = 5) and lure types are treatments. Separate analyses were performed on mean season-long cumulative catches of males, females, and total catches for both sterile and wild moths using Tukey’s test. Experiment 3 was analysed using a randomised block ANOVA, but in this analysis orchards were used as replicate blocks (*n* = 4). Catches with each lure treatment from the three sets within each orchard were summed to give a single datum for each lure treatment in each orchard.

Contingency tables and chi-square (χ^2^) tests were used to compare the ratios of sterile (S) and wild (W) moths caught by various lures in each experiment. The equality of these S:W ratios (i.e., moth catch frequencies) across lure types under different management regimes (i.e., SIT or SIT + MD) was tested under the null hypotheses of equal frequencies across lures and/or independence from orchard management regime [25]. Seasonal total catches with each type of lure within an orchard treatment (SIT or SIT + MD) were used to calculate S:W ratios as is standard for the Canadian SIR Program [12]. All statistical analyses were performed with experimental error rates set at α = 0.05 using SigmaPlot^®^ 12.5 (SYSTAT Software Inc., San Jose, CA, USA).

## 3. Results

### 3.1. Experiment 1

The relative catches of sterile and wild codling moths with different kairomone lures appear similar in orchards managed by SIT (Table 1). For each moth sex and type (sterile and wild), the AA-PE combination lure was the most attractive kairomone lure tested in experiment 1 (Table 1). PE was numerically the most attractive single component for wild male and female moths, but statistically there was no separation of catches with PE and AA for wild females (Table 1). On a relative basis, AA appeared more attractive to sterile moths than it did wild moths, catching as many sterile moths as did PE in this test (Table 1).

In this experiment, season-long mean cumulative catch of sterile males in four 1 mg codlemone-baited wing traps operated by the SIR Program was 413.8 ± 28.3 moths. This seasonal catch is about 2.7× more than the number of sterile males caught with AA-PE lures (Table 1). In notable contrast, season-long mean cumulative catch of wild male codling moths in these same SIR Program codlemone-baited traps was only 4.5 ± 1.7, less than half the number of wild males caught (11.3 ± 4.1) with four AA-PE lures in the same orchard (Table 1).

**Figure 1:**
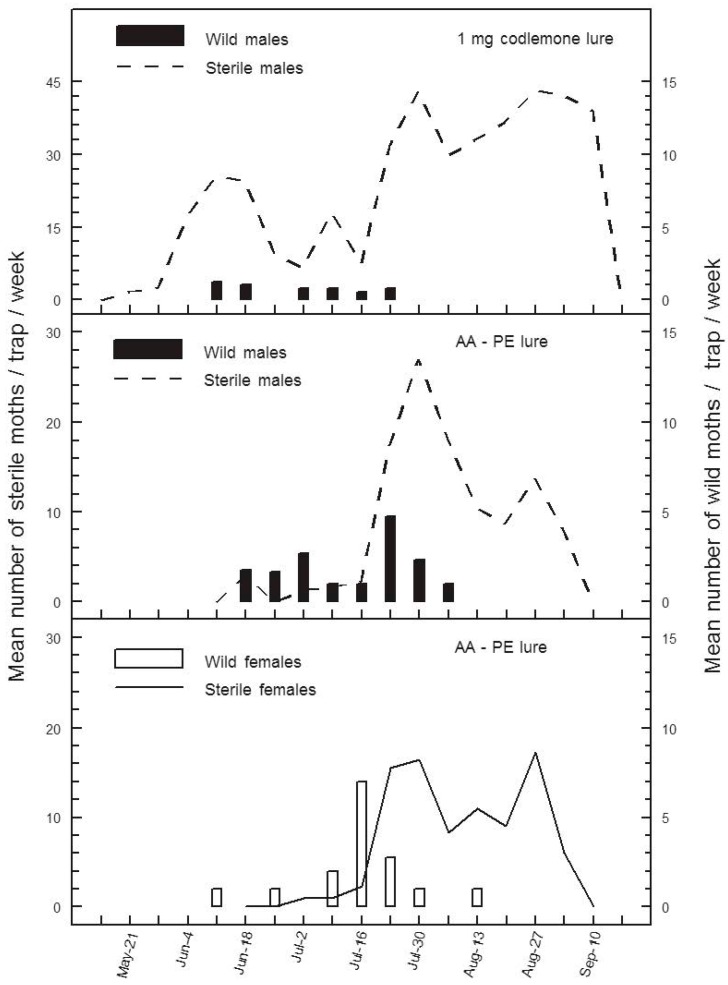
Mean weekly catches of sterile and wild male codling moths in wing traps baited with a 1 mg codlemone-loaded red rubber septum, and catches of male and female, sterile and wild codling moths in white delta traps baited with a propylene bottle containing 10 mL of glacial acetic acid plus a grey rubber septum loaded with 1 mg of pear ester, ethyl-(*E*,*Z*)-2,4-decadienoate (AA-PE) in 2008.

Seasonal activity of wild moths measured by weekly catches with pheromone traps was somewhat different from that reflected by AA-PE lures (Figure 1). Peak mean weekly catches of wild male (4.8) and female (7.0) codling moths with AA-PE lures occurred on 24 and 16 July 2008, respectively, a time when few wild males (0–0.8 moths/week) were caught in SIR Program pheromone traps (Figure 1). The S:W ratios for male (0.27:1) versus female (0.32:1) moths based on total catch (*n* = 4 traps) with AA-PE lures at peak wild catch were not significantly different (χ^2^ = 0.071, df = 1, *p* = 0.896), but both were well below the male S:W ratio (43:1) estimated with SIR Program pheromone traps from 16 to 24 July (Figure 1). Seasonal male S:W ratios measured with codlemone lures (92:1) were skewed even further by large late-season catches of sterile males when wild males were absent (Figure 1). Catches of wild males with codlemone lures went to zero as catches of sterile moths increased in all trap types. Wild codling moth catches with AA-PE lures continued for two weeks after wild males ceased being caught in pheromone traps (Figure 1).

### 3.2. Experiment 2

Catches of sterile and wild codling moths with PE combination lures and standard pheromone lures used by the SIR Program in apple orchards managed by SIT and SIT + MD are compared in Table 2. CM-DA lures caught significantly more sterile males than did AA-PE lures in areas under management by SIT or SIT + MD, whereas AA-PE lures caught significantly more sterile females than CM-DA lures under either management regime (Table 2). Catches of wild moths were small in experiment 2 and there were no significant differences among lures for either sex under either management regime (Table 2).

**Figure 2:**
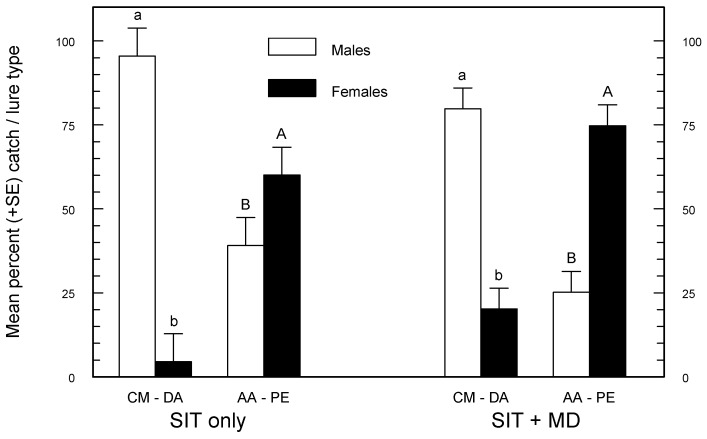
Mean (±SE) percentage of male and female sterile codling moths caught in white delta traps baited with either a single 8 mL propylene bottle containing two small cotton balls with a 3 mm diameter hole in its lid and loaded with 5 mL of an acetic acid plus PE mixture (240:1 µL *v:v*) (AA-PE), or a grey rubber septum loaded with 3 mg of codlemone and 3 mg of ethyl-(*E*,*Z*)-2,4-decadienoate (CM-DA) in an orchard receiving sterile moths only (SIT) and one receiving sterile moths combined with an Isomate-CM/LR-TT pheromone mating-disruption treatment (SIT + MD). Paired bars within an orchard and lure treatment having different letter superscripts are significantly different by paired *t*-tests (*p* < 0.05).

In terms of total sterile moth catch, the CM-DA lure caught 2.7 and 2.1× more sterile moths than the AA-PE lure in areas managed by SIT or SIT + MD, respectively (Table 2). However, under both management systems, the seasonal total sterile moth catch with the AA-PE lure had a significantly greater percentage of females, and the CM-DA lure had a significantly greater percentage of males (Figure 2).

**Figure 3:**
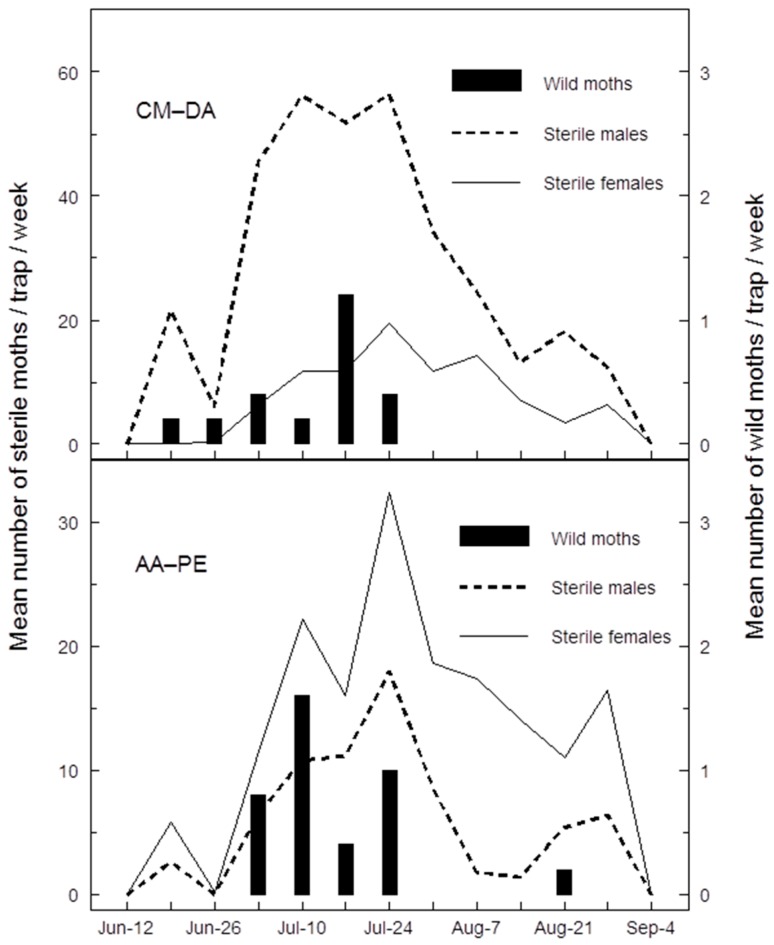
Mean weekly catches of male and female, sterile and wild codling moths in white delta traps baited with either a single 8 mL propylene bottle containing two small cotton balls with a 3 mm diameter hole in its lid and loaded with 5 mL of an acetic acid plus PE mixture (240:1 µL *v:v*) (AA-PE), or a grey rubber septum loaded with 3 mg of codlemone and 3 mg of ethyl-(*E*,*Z*)-2,4-decadienoate (CM-DA) in an area under management by SIT + MD (Isomate-CM/LR-TT) in 2009.

Both CM-DA and AA-PE lures revealed seasonal variation in catches of sterile moths, even though sterile moths were delivered at a constant weekly rate (4000 mixed-sex·ha^−1^) from early May through August. Small numbers of each sterile moth sex were recaptured in June, maximum catches occurred throughout July, and catches declined in August (Figure 3). The sex ratio in weekly catches of sterile moths was notably reversed with the two different PE lures. Weekly sterile catches with CM-DA lures had a greater percentage of males, whereas catches with AA-PE lure had a greater percentage of females (Figure 3).

Although catches of wild moths were small, when weekly catches of wild male and female moths were pooled it was possible to see seasonal patterns with both CM-DA and AA-PE lures in an area managed by SIT + MD (Figure 3). Catches of wild moths with CM-DA and AA-PE lures were separated temporally from each other (Figure 3). First wild catch with CM-DA lures occurred on 19 June, while wild catches with AA-PE lures started on 3 July. Wild catches with CM-DA ended 24 July, but last catch with AA-PE lures occurred almost three weeks later on 21 August 2009 (Figure 3). Dates of peak mean weekly total wild catches with the two lures were one week apart, occurring on 17 July with CM-DA lures and on 10 July 2009 with AA-PE lures. Although the maximum mean weekly wild catches with CM-DA (1.2) and AA-PE lures (1.6) were not different (Figure 3), the S:W ratios based on total catches (*n* = 5 traps) at peak wild catch with CM-DA (53:1) and AA-PE (21:1) lures were significantly different (χ^2^ = 5.361, df = 1, *p =* 0.021). This difference occurs in part because catches of wild female moths peaked earlier than catches of sterile female moths, and females make up most of the wild catch with AA-PE lures (Figure 3).

### 3.3. Experiment 3

Relative catches of sterile and wild codling moths with PE combination lures in replicated orchards managed by SIT or SIT + MD in experiment 3 (Table 3) were similar to those in experiment 2 (Table 2). In orchards under SIT management, CM-DA lures caught significantly more sterile males than all other lures, sterile male catches declined as codlemone load decreased, and AA-PE lures caught the fewest sterile males (Table 3). In these same SIT-managed orchards, significantly more sterile females were caught with AA-PE lures than with CM-DA lures (Table 3).

In orchards under management by SIT + MD, the CM-DA lures caught the greatest number of sterile males, catches of sterile males decreased as codlemone load decreased, and catches of sterile males using AA-PE lures were not significantly different than catches of sterile males with a 0.1 mg codlemone lure (Table 3). AA-PE lures caught more sterile females than CM-DA lures in orchards receiving SIT + MD. In the SIT + MD orchards, sterile females made up 81% of the total sterile moth catch using AA-PE, but only 15% of total sterile catch with CM-DA lures (Table 3).

Although there were no statistical differences among catches of wilds with individual lures in orchards under management by SIT (Table 3), the ranked order of wild male and female catches with different lures was in general concordance with those of sterile moths in the same orchards, respectively. In orchards managed by SIT + MD, in experiment 3, relative catches of wild males mirrored those of sterile males (Table 3). CM-DA lures caught significantly more wild males than all other lures, catches of wild males decreased with decreasing codlemone dose, and AA-PE lures caught a similar number of wild males to that of the 0.1 mg codlemone lure. Catches of wild females with both AA-PE and CM-DA lures were small and not significantly different in orchards managed by SIT + MD (Table 3). In orchards managed by SIT + MD, wild females made up 83% of the total wild catch with AA-PE lures but only 13% of the total wild moth catch with CM-DA lures (Table 3).

### 3.4. Sterile:Wild Ratios by Experiment, Lure, Background and Moth Sex

S:W ratios for each moth sex separately were calculated using seasonal total sterile and wild moth catches using AA-PE versus CM-DA lures in orchards managed by SIT or SIT + MD in experiments 2 and 3 (Table 4). Within each experiment, S:W ratios estimated using male versus female moth catches with AA-PE lures were not significantly different, whereas catches with CM-DA lures generated highly variable—and most often significantly different—male versus female S:W ratios (Table 4).

S:W ratios measured using AA-PE lures were independent of the orchard management programme. In both experiments 2 and 3, there were no significant differences between the male versus female S:W ratios using AA-PE lures in orchards managed by SIT or SIT + MD, respectively (Table 4). In contrast, male versus female S:W ratios estimated from catches with CM-DA lures in orchards managed by SIT were significantly different in experiments 2 and 3 (Table 4). These differences reflect disproportionate catches of sterile males with these lures (Table 2 and Table 3). Male S:W ratios calculated from catches using CM-DA lures were always significantly different than male S:W ratios estimated using AA-PE lures (Table 4). Female S:W ratios with CM-DA lures were inconsistent but mostly similar to female S:W ratios with AA-PE lures under either orchard management regime (Table 4).

## 4. Discussion

Monitoring seasonal phenology and tracking densities of sterile moths in relation to that of wild moths is critical in codling moth SIT programmes. Wild catches are used to recommend supplemental controls, and resulting S:W ratios can be used to direct programme staff where to increase or decrease the number of sterile moths being delivered [12]. It was logical for a codling moth SIT programme implemented in 1992 to use pheromone traps to estimate operational S:W ratios [3]; at the time there was no reasonable alternative, and pheromone traps had a successful history in codling moth pest management [8]. As soon as the SIR Program began, it became clear that area-wide male S:W ratios were often poor predictors of population control in individual orchards [5,12]. The need to estimate local S:W ratios, in contrast to regional or global ratios for the programme as a whole, was particularly important in understanding the slow progress of the Canadian SIR Program [12]. The apple industry in BC consists of many small, but contiguous, properties. The SIR Program has had difficulty correlating summary S:W ratios (as estimated with pheromone traps) with damage in specific legal properties or parts thereof. The current SIR Program mostly ignores S:W ratios and advises growers to apply insecticides and/or MD as supplemental controls based on total wild counts (personal communication). Improved accuracy in measuring S:W ratios may allow the SIR Program to be more responsive to local orchard needs and encourage greater precision in delivering sterile moths when and where needed.

Problems in estimating S:W ratios have probably arisen in part because wild codling moth populations are generally aggregated with respect to their overwintering and subsequent spring emergence sites [26], whereas sterile moths are uniformly distributed everywhere. The theory behind the original SIR Program protocols and effectiveness assumed that spatial structure of the moth population could be ignored [3]. However, more recent modelling studies have shown that it can have a profound effect on control and trapping [13]. Spatial differences in codling moth population structure likely leads to localised, within-orchard differences in S:W ratios. Uniform delivery of sterile moths into an orchard moth population at or above the 40:1 ratio may well result in S:W ratios that are below the 40:1 target in areas where wild moths are aggregated. This is because the sterile moth-control effort (the 40 sterile moths) is distributed and diluted over the entire orchard area, whereas wild moths are concentrated within small sections of orchards. What matters most, in terms of control by SIT, is that S:W ratios are consistently at or above the 40:1 threshold when and where wild moths are present (i.e., in these spring aggregations). This is because codling moth has a scramble competition mating system, and the nearest and fastest responding males are the ones most likely to find and mate the wild female when she releases pheromone [13,14]. Measuring and then maintaining appropriate S:W ratios in these areas of aggregation are key to early-season control by SIT. Without ancillary information about population structure, catches of wild moths in spring are the best indicators of these sites of aggregation, because cool temperatures often keep moths near their emergence sites. It follows that sampling sterile moth populations in close proximity to these wild detections will more accurately measure ratios within or near wild aggregations.

Standard codlemone lures used to measure codling moth S:W ratios may attract moths from more than 100 m [27], so the moths caught might emerge in close proximity to the trap location or at some distance away. Because sterile moths are everywhere, while wild moths are not, differences in the spatial distribution of sterile and wild moths changes their respective probabilities of capture. All else being equal, the chances of catching wild moths in randomly distributed codlemone-baited traps is significantly lower than that of sterile moths because the dispersion of wild moths within orchards is aggregated, while the distribution of sterile moths is even throughout orchards. Codlemone-baited traps that happen to catch a nearby wild male will not only attract nearby sterile males, but also those up to at least 100 m away. Sterile and wild males separated by many meters are likely not actively competing for the same mates [13,14]. Therefore, codlemone lures that sample and attract sterile males from surrounding areas into an area where a wild male is caught may accurately estimate S:W ratios within the larger orchard population, but will likely overestimate ratios within or near the origin of the wild male. On the other hand, a short-range lure that attracts a nearby wild male should only sample and attract sterile males in close proximity to that wild male and, therefore, it should measure local S:W ratios more accurately than a long-range lure. It may also measure female S:W mating ratios more accurately when wild males and females are aggregated.

Plant volatile kairomones are generally thought to have sampling ranges of perhaps 5–10 m [28,29,30]. Given this fact, and the argument that short-range lures have a higher probability of estimating S:W ratios near the site a wild insect is caught, AA-PE lures should offer greater chances than pheromone lures of measuring localised S:W ratios and correlating codling moth trap catches with damage in localised areas. Furthermore, because wild codling moth populations are aggregated, and these aggregations normally have 1:1 sex ratios, and sterile moths are delivered in a 1:1: sex ratio [14], short-range lures that catch male and female codling moths should be expected to yield the same S:W ratios for male or female moth catches, providing sterile and wild moths respond to lures similarly. Given that there is a the lack of evidence that the respective responses of male and female sterile codling moths to AA-PE lures are different than their wild counterparts (Table 1), the fact that male versus female S:W ratios in catches with AA-PE lures were identical (Table 4) meets the expectation that AA-PE is a short-range lure for both sexes. Moreover, AA-PE is likely a short-range lure in orchards managed by SIT or SIT + MD, because male versus female S:W ratios in catches with AA-PE lures were not significantly different under these different management regimes (Table 4).

Although CM-DA lures also catch both males and females, catches with these lures yielded male S:W ratios that were significantly greater than female S:W ratios in 3 of 4 cases (Table 4). This probably occurs because the pheromone component of the CM-DA lure acts to attract males over the long range, while the PE component likely attracts females over a shorter range. This would explain why male S:W ratios estimated using CM-DA lures in orchards managed by SIT were similar to those estimated with codlemone lures, even those with reduced loads (Table 3). In orchards managed by SIT + MD, where the attraction range of a codlemone-based lure is arguably shortened by the background of MD pheromone, all codlemone-based lures produced male S:W ratios more similar to that of an AA-PE lure than they did in orchards managed by SIT (Table 3).

CM-DA lures provided the same estimates of female S:W ratios as did AA-PE lures in the same orchards on 2 of 4 occasions (Table 4). This suggests that the amounts of PE in each lure, although different, were probably acting over the same distances for females and providing the same female S:W ratios. Nevertheless, CM-DA lures would not be useful for measuring female S:W ratios, because identifying female moths among so many males (Table 2 and Table 3) was prohibitively time consuming. On the other hand, traps baited with AA-PE lures caught 1.7× more females than CM-DA lures; more importantly, they caught 95% fewer males than did traps with CM-DA lures (Table 2 and Table 3). Small total catches with AA-PE lures makes it possible to consider sexing moths, but this would seem unnecessary because S:W ratios based on male or female catches were not significantly different using AA-PE lures (Table 4). It appears that the commercial CM-DA lures provide little improvement over standard 1 or 10 mg codlemone lures for estimating S:W ratios in orchards managed by SIT or SIT + MD, respectively. CM-DA lures, like all codlemone lures (even those with low loads (Table 3)), likely overestimate localised S:W ratios and the impact of the SIT control effort [13,14].

If maximising catches of male moths was the objective of a codling moth monitoring programme, then data presented here (Table 2 and Table 3) suggest CM-DA lures are the best choice in orchards managed by SIT or SIT + MD. However, catching large numbers of sterile males in an SIT programme may be detrimental to the control effort, because the males caught may represent the strongest and most competitive males released. Furthermore, catching more sterile moths increases the work load of trap monitors and may reduce detection of wild moths. In summer, pheromone-baited wing traps used by the SIR Program often become saturated with sterile males, averaging over 35 moths/trap/week during experiment 1, but individual traps often have twice this many in summer (SIR Program; www.sir.org). When large numbers of sterile moths are caught, it becomes difficult for SIR Program staff to detect wild moths among so many sterile moths because traps are serviced quickly in the field. An ideal monitoring lure for an SIT programme may be one that minimises the number of functional sterile males removed from the mating population while simultaneously tracking wild population activity and providing robust estimates of S:W ratios.

Both AA-PE and CM-DA lures appear to track the seasonal phenology of wild codling moth at extremely low population densities, apparently sometimes better than pheromone lures in orchards managed by SIT or SIT + MD, respectively (Figure 1 and Figure 3). For example, in experiment 1, the number of season-long catches of wild male codling moths in codlemone-baited traps was less than half the number of wilds caught with AA-PE lures (Table 1). Furthermore, catches of wild male codling moths were low or undetectable in pheromone traps at times when catches of wild codling moths were at their peaks using AA-PE lures (Figure 1). This discrepancy and obvious late-July to August decline in catches of wild males in pheromone traps may have something to do with changes in the activity of sterile females. Seasonal variation in catches of sterile males in pheromone traps is a well-documented phenomenon in the Canadian SIR Program [12]; catches with AA-PE lures have provided the first evidence that sterile females have the same seasonal activity as sterile males (Figure 1). An increase in catches of sterile moths in summer likely reflects their increased flight activity at warmer temperatures [31]. Warmer weather likely increases the activity of unmated sterile females and the number releasing sex pheromone (calling). The efficiency of codling moth pheromone traps is reduced by competition between calling females and the trap lure [32]; therefore, in experiment 1, competition between calling sterile females and pheromone traps may have reached a peak in late July, when standard pheromone traps ceased catching wild males (Figure 1). Throughout this same period, AA-PE lures continued to catch both male and female wild codling moths probably because kairomones do not compete with natural or synthetic pheromone sources.

The Canadian SIR Program continues to combine SIT and MD to meet its objective. Monitoring populations of codling moth in areas under control by MD or SIT presents different yet similar challenges. Both management systems reduce the efficiency of pheromone traps, because trap lures compete with pheromone sources that are either thousands of times more concentrated (MD dispensers) or more numerous (in the form of virgin sterile females released jointly with sterile males [3]). Standard 1 mg codlemone lures are rendered less effective for monitoring codling moth under MD programmes (Table 2 and Table 3). The solution has been to use higher load codlemone lures [9,24] and, more recently, CM-DA lures (codlemone–PE lures) [22]. Both AA-PE and CM-DA lures perform better than standard pheromone lures at tracking seasonal phenology or catching codling moths in orchards treated with MD (Table 2 and Table 3). Therefore, choosing between these two PE combination lures may depend on whether the target of monitoring is male or female codling moth. CM-DA lures caught a significantly greater percentage of males, and AA-PE caught a significantly greater percentage of females, independent of the background treatment (Figure 2). These results are in close agreement with Knight [21] who found that 91%–99% of total wild codling moth catch was male in orange delta traps baited with CM-DA lures, and 43%–71% of the catch was female when clear delta traps were baited with AA-PE lures. Theoretically, it should be easier to predict potential damage from female density, and AA-PE lures are best for measuring this. Furthermore, AA-PE lures have an advantage over CM-DA lures because male, female, and male + female S:W ratios derived from catches with the AA-PE lure were more consistent and independent of the management regime (Table 4). There are obvious operational advantages if a single lure can be used by the SIR Program under all management situations; AA-PE lures make this possible.

The Okanagan–Kootenay SIR Program was a catalyst for a large international effort to develop improved techniques and expand the use of SIT for management of codling moth [33]. While that expansion is under way in South Africa, Argentina, and, most recently, New Zealand [34,35] (www.sir.org), these pilot programmes (like the Canadian SIR Program), depend almost exclusively on field methods developed before 1980 [3,11]. There have been a myriad of studies to improve monitoring of codling moth since this Canadian SIR Program began, yet the most recent review [33] failed to mention any development of new tools to monitor or measure codling moth S:W ratios in operational programmes. In over 40 years, there have been no improvements in estimating and accurately measuring what is clearly the foundation of a successful codling moth SIT programme. AA-PE lures offer an opportunity to change that and, in doing so, answer some interesting fundamental questions regarding management of codling moth with SIT.

In an operational SIT programme, reduced trap catches using AA-PE lures have the advantage of saving field staff time servicing individual traps that may indirectly improve the probability of detecting a wild moth. Small catches with AA-PE lures mean fewer functional sterile males are removed by each trap, which perhaps allows for use of more traps and fine-grained monitoring of S:W ratios with no negative effects. AA-PE lures may even promote use and development of labour-saving automated trapping systems [36,37] that visually discriminate between wild and sterile moths based on external fluorescent markings [14]. The ability of AA-PE lures to capture a high percentage of females is a great advantage to SIT because it enables monitoring of female activity and development of more accurate female-based action thresholds [21]. Given high female capture with AA-PE lures—even greater when placed in clear delta traps [20]—it should be possible to accurately determine the percentage of females that are mating under different management programmes. Combining this information with tools for analysing the origin of a spermatophore [38] might make it possible to determine what type of male the wild (and even sterile) females are mating with, and in what frequency. This information is important, because the extent to which sterile females serve as a sink for wild sperm, and how this affects control in a codling moth SIT programme that is releasing both sexes, is an unresolved question. Practitioners of codling moth SIT have long pondered separating the sexes before releasing moths on the assumption that control might be better if sterile males were released alone.

## 5. Conclusions

Use of AA-PE lures warrants an expanded evaluation as an operational method for tracking S:W overflooding ratios in the Canadian SIR Program and as a research tool to more directly measure mating success and control effort in developing new codling moth SIT programmes. Future research should evaluate the reliability of commercially available AA-PE lures, develop and evaluate female thresholds, and seek to optimise trap density. More AA-PE-baited traps may be needed to ensure their shorter range of activity, compared with codlemone-baited traps, does not compromise detection of wild populations and damage, at the expense of providing improved measurement of reliable S:W ratios.

## Figures and Tables

**Table 1:** Comparative seasonal catches of sterile and wild codling moths in white delta traps baited with acetic acid (AA) or ethyl-(*E*,*Z*)-2,4-decadienoate (pear ester (PE)) alone and in combination (AA-PE) in an apple orchard receiving weekly delivery of sterile moths.

Moth Sex	Lure Type ^1^	Mean (±SE) Cumulative Number of Moths Caught/Trap ^2,3^		
		Sterile	Wild	Total
Male	AA-PE	151.5 ± 42.0 a	11.3 ± 4.1 a	162.8 ± 26.5 a
	AA	51.3 ± 54.0 b	0.8 ± 0.7 c	52.0 ± 31.6 b
	PE	35.3 ± 25.3 b	6.8 ± 7.1 b	42.0 ± 18.0 b
	Blank	0.8 ± 1.3 c	0.0 ± 0.0	0.8 ± 1.3 c
Female	AA-PE	77.0 ± 23.4 a	12.8 ± 2.9 a	89.8 ± 13.2 a
	AA	17.8 ± 13.1 b	1.3 ± 1.1 b	19.0 ± 17.1 b
	PE	18.5 ± 16.1 b	9.3 ± 6.4 b	27.8 ± 12.9 b
	Blank	0.6 ± 1.2 c	0.0 ± 0.0	0.6 ± 1.2 c
Totals	AA-PE	228.5 ± 114.3 a	24.0 ± 1.3 a	252.5 ± 37.8 a
	AA	69.1 ± 33.0 b	2.0 ± 0.7 b	71.0 ± 30.7 b
	PE	53.8 ± 20.5 b	16.0 ± 7.7 a	69.8 ± 30.3 b
	Blank	1.4 ± 1.2 c	0.0 ± 0.0	1.4 ± 1.2 c

^1^ AA lure is a 15 mL propylene bottle containing 10 mL of glacial acetic acid released through a 3 mm hole in the lid; PE lure is a grey rubber septum loaded with 1 mg of ethyl-(*E*,*Z*)-2,4-decadienoate and 10 mg of butylated hydroxytoluene (BHT) antioxidant; AA-PE lure is a combination of the two lures. ^2^ Means (*n* = 4 traps) within a column for a given moth sex or total sterile and wild moths, followed by different letters are significantly different (Tukey’s test, α = 0.05) following a significant (*p* ≤ 0.05) Latin square ANOVA. Treatments catching zero moths were excluded from statistical tests. ^3^ Test orchard received one weekly delivery of 4000 mixed-sex (1:1) sterile codling moths ha^−1^.

**Table 2:** Comparative seasonal catches of sterile and wild codling moths in white delta traps baited with various lures in a 17 ha area of apple orchards receiving a sterile insect treatment (SIT) alone, and in an adjacent 12.5 ha area receiving an SIT treatment combined with a pheromone mating-disruption (MD) treatment.

Orchard Treatment ^1^	Lure Type ^2^	Mean (±SE) Cumulative Number of Moths/Trap ^3^			
		Males		Females	
		Sterile	Wild	Sterile	Wild
SIT	AA-PE	118.2 ± 11.3 b	1.2 ± 0.4 a	144.2 ± 34.4 a	1.6 ± 0.7 a
	CM-DA	649.0 ± 55.5 a	2.4 ± 0.9 a	62.6 ± 14.9 b	3.0 ± 1.6 a
	1 mg codlemone	603.8 ± 35.7 a	1.8 ± 0.9 a		
SIT + MD	AA-PE	126.0 ± 25.3 c	1.4 ± 0.7 a	224.6 ± 30.9 a	2.6 ± 0.4 a
	CM-DA	603.6 ± 72.5 a	1.4 ± 0.4 a	125.8 ± 14.3 b	1.2 ± 0.5 a
	10 mg codlemone	409.8 ± 65.7 b	0.4 ± 0.2 a		

^1^ SIT treatment was one weekly delivery of 4000 mixed-sex (1:1) sterile codling moths ha^−1^; MD treatment was 750 Isomate-CM/LR-TT dispensers ha^−1^. ^2^ AA-PE lure is a single 8 mL propylene bottle with a 3 mm diameter hole in its lid containing two small cotton balls and a 5 mL load of an acetic acid plus PE mixture (240:1 µL *v:v*); CM-DA lure is a grey rubber septum loaded with 3 mg of codlemone *plus* 3 mg of pear ester; codlemone loaded on grey rubber septum. ^3^ Means (*n* = 5 traps/area) in a column for a given orchard treatment and moth sex followed by different letters area significantly different (Tukey’s test, α = 0.05) following a significant (*p* ≤ 0.05) randomised block ANOVA.

**Table 3:** Comparative seasonal catches of sterile and wild codling moths in white delta traps baited with various lures in apple orchards under management by sterile insect treatment (SIT) alone or a combination of SIT and pheromone mating-disruption (MD).

Orchard Treatment ^1^	Lure Type ^2^	Mean (±SE) Cumulative Number of Moths/Trap ^3^			
		Males		Female	
		Sterile	Wild	Sterile	Wild
SIT	AA-PE	40.7 ± 13.1 d	1.0 ± 0.5 a	54.3 ± 8.6 a	2.0 ± 0.7 a
	CM-DA	699.0 ± 68.6 a	3.7 ± 1.3 a	32.6 ± 7.6 b	1.1 ± 0.4 a
	1.0 mg codlemone	470.6 ± 47.0 b	2.0 ± 0.8 a		
	0.1 mg codlemone	336.3 ± 43.7 c	1.1 ± 0.9 a		
SIT + MD	AA-PE	9.3 ± 0.9 c	0.4 ± 0.3 c	39.1 ± 5.3 a	3.0 ± 1.0 a
	CM-DA	132.6 ± 23.6 a	19.7 ± 9.5 a	22.6 ± 2.5 b	2.0 ± 0.6 a
	10.0 mg codlemone	100.1 ± 13.3 a	7.6 ± 2.8 b		
	1.0 mg codlemone	37.0 ± 7.7 b	2.9 ± 1.5 bc		
	0.1 mg codlemone	4.9 ± 1.4 c	0.1 ± 0.1 c		

^1^ SIT treatment was one weekly delivery of 4000 mixed-sex (1:1) sterile codling moths ha^−1^; MD treatment was 1000 Isomate-CM-Flex dispensers ha^−1^. ^2^ AA-PE lure was a single 8 mL propylene bottle with a 3 mm diameter hole in its lid containing two small cotton balls and a 5 mL load of an acetic acid plus PE mixture (240:1 µL *v:v*); CM-DA lure is a grey rubber septum loaded with 3 mg of codlemone *plus* 3 mg of pear ester (Trécé); codlemone loaded on grey rubber septa. ^3^ Means (*n* = 4) within a column and orchard treatment followed by different letters are significantly different (Tukey’s test, α = 0.05) following a significant (*p* ≤ 0.05) randomised block ANOVA.

**Table 4:** Total seasonal catch of sterile (S) and wild (W) codling moths with acetic acid *plus* pear ester (AA-PE) and codlemone *plus* pear ester (CM-DA) lures in delta traps operated within orchards under management by sterile insect treatment (SIT) alone or combined with pheromone mating-disruption (MD).

Experiment Number	Orchard Treatment ^1^	Lure ^2^	Males ^3^		Females ^3^		S:W Ratio		Contingency Table Analysis of Male Versus Female S:W Ratios within a Row (Lure)	
			Sterile	Wild	Sterile	Wild	Male	Female	χ^2^ statistic	*p*-Value
Exp. 2	SIT	AA-PE	579	6	720	8	99:1	90:1	0.0201	0.887 ns
		CM-DA	3467	12	313	15	270:1	21:1	64.9711	0.001 **
			χ^2^ = 3.847 df = 1, *p =* 0.05		χ^2^ = 11.232 df = 1, *p =* 0.001					
						
	SIT + MD	AA-PE	630	7	1123	13	90:1	86:1	0.0217	0.883 ns
		CM-DA	3019	7	629	6	432:1	105:1	5.6711	0.017 **
			χ^2^ = 8.249 df = 1, *p =* 0.004		χ^2^ = 0.226 df = 1, *p* = 0.881					
						
Exp. 3	SIT	AA-PE	379	9	489	18	42:1	27:1	0.756	0.385 ns
		CM-DA	6291	33	293	10	191:1	29:1	30.451	0.001 **
			χ^2^ = 16.219 df = 1, *p* = 0.001		χ^2^ = 0.0001 df = 1, *p* = 0.992					
						
	SIT + MD	AA-PE	133	5	388	19	27:1	20:1	0.0768	0.782 ns
		CM-DA	1734	245	355	56	7:1	6:1	0.3730	0.541 ns
			χ^2^ = 8.676 df = 1, *p* = 0.003		χ^2^ = 18.639 df = 1, *p* = 0.001					
						

^1^ SIT treatment was one weekly delivery of 4000 mixed-sex (1:1) sterile codling moths ha^−1^; MD treatment was 750 Isomate-CM/LR TT dispensers ha^−1^ in Exp. 2 and 1000 Isomate-CM-Flex dispensers ha^−1^ in Exp. 3.

^2^ AA-PE lure was a single 8 mL propylene bottle with a 3 mm diameter hole in its lid containing two small cotton balls and a 5 mL load of an acetic acid plus PE mixture (240:1 µL *v:v*); CM-DA lure is a grey rubber septum loaded with 3 mg of codlemone plus 3 mg of pear ester.

^3^ Results of χ^2^ analyses are shown beneath each 2 × 2 contingency table of sterile and wild moth catches for each sex to test null hypothesis that the two lures provide the same S:W ratios within each orchard management treatment and experiment.

## Abbreviations

The following abbreviations are used in this manuscript:

MD	Mating disruption
SIT	Sterile Insect Technology
SIR	Sterile Insect Release
S	Sterile moths
W	Wild moths
SW	Sterile to wild moth ratio
AA	Acetic acid
PE	Pear ester
AA-PE	Acetic acid plus pear ester lure
CM-DA	Codlemone plus pear ester lure
ATV	All-terrain vehicle
